# Genetics of autism and mental retardation: A spoonful from the sea!

**DOI:** 10.4103/0971-6866.60181

**Published:** 2009

**Authors:** Kanjaksha Ghosh, Ajit Gorakshakar

**Affiliations:** National Institute of Immunohaematology, K.E.M Hospital Campus, Parel, Mumbai, India

Intellectual disability comprises wider spectrum of conditions other than mental retardation. Defining features of mental retardation includes an IQ below 70 and significant limitations in two or more adaptive skills, and autism has other defining features but mental retardation can be part of this condition in 50–70% of the cases.[1] Although we do not know how many mentally retarded children are born in this country and what is the social, financial, and emotional cost of this condition in our country, we know very well that Down's syndrome and Fragile X syndrome are two common causes of mental retardation and can easily be diagnosed during gestation. However, the number of pregnant mothers availing this facility is not encouraging, and moreover there are not enough centers in our country where a simple karyotype and high-resolution banding can be done.

We should not forget that though in a large number of children with autism no cause could be detected (80%) and only 5–10% of the autism patients are secondary, but only 3% of the patient have inherited chromosomal anomaly.[2] Recently, a reciprocal microduplication or recurrent microdeletion at 16p 11.2 has been shown to be associated with autism and may account for 1% of the cases.[3]

However, mental retardation (intellectual disability) is also common and wherever studied is responsible for 1–3% general population.[4] Mental retardation can be associated with innumerable factors – some are chromosomal, some are genetic, some are metabolic, and some are due to intrauterine infections, and where antenatal and natal care is not adequate a large number of them may be due to birth asphyxia or other accidents during peripartum period. Mental retardation due to metabolic disorders like congenital phenylketonuria is preventable. If hypothyroidism is diagnosed early, mental retardation from this condition is also completely preventable, and in many countries there is a neonatal screening program for common biochemical abnormalities leading to mental retardation. In this country, Indian council of Medical Research did an extensive study almost 20 years back[5] and is again indulging on similar study, but in the intervening period nothing substantial has been done by different states or central government to mitigate this needless suffering. We can only hope that recent ongoing study may result in more desirable steps taken by the caregivers of the country.

A large number of mental retardation is due to cryptic or overt chromosomal abnormalities, which can only be detected by modern cytogenetic techniques which may involve in addition to standard cytogenetics, subtelomeric FISH, centromeric FISH, array-CGH to name a few.

However, proper selection of patients through clinical features, pedigree chart, and certain biochemical analysis (abnormally high free triidothyronine in SLC *16A2* gene mutation, abnormal urine, and plasma creatine/creatinine ratio in SLC6A8 mutation) allows proper use of these genetic tools.

A large-scale cytogenetic study from special schools in Taiwan between 1991 and 1996 involving 11,892 children showed that genetic cause accounted for 38.5% cases of MR (intellectual disability). Serious disability was seen in 37% of the cases. Perinatal and postnatal cause was found to be operative in 13% of the patients.[6] These patients probably would have been normal if natal and prenatal care were good. It is quite well known that syndromic causes of mental retardation often give a clue to the nature of chromosomal defect and allow the clinician to go for prenatal diagnosis. However in nonsyndromic cases, the genetic and chromosomal abnormality may not be so easily discernable.[7] In our country, clinical geneticists are rare even in the best of the medical institutes. This is a significant health handicap in a country of 1 billion people where the number of persons born with mental retardation and other genetic disabilities are likely to be substantial.

When in a family a baby with mental retardation is born, the family members want to know:

- Is this mental retardation genetic?
- What is the risk of having similar mental retardation in the subsequent pregnancy?
- How to manage the baby?
- Can we prevent occurrence of future birth of similarly attached baby through preconception genetic diagnosis or prenatal diagnosis?

As discussed in cases of known chromosomal aberrations (gross, microdeletion/duplication either subtelomeric or interstitial), this may be possible. Same is also true if the single gene mutation (autosomal recessive, autosomal dominant, or X linked) in causing the mental retardation and the mutation is well characterized. Unfortunately this is not always possible. Moreover there is a proportion of inherited mental retardation which is multigenic and involves epigenetic modification of the genome.

We have not yet understood all the genetic factors involved in mental retardation. Currently more than 50 genes have been associated with syndromic and 25 genes with nonsyndromic X-linked mental retardation only and this is expected to be only 40% of the families with X-linked mental retardation.[8] However, very few autosomes have been identified as a cause of autosomal recessive mental retardation. Hence genetic heterogeneity in mental retardation is substantial and we have only started scratching the surface of the problem. In the present issue of this journal, Bhanumathi *et al*'s article[9] should be analyzed from the above mentioned perspective. Presently we can only identify genes, chromosomal changes, some microdetections fragile X, to name a few.

So what should be the present strategy of investigating a child with mental retardation, with or without autism?

- A careful clinical evaluation with specific reference to dysmorphology or other phenotypic abnormalities has to be made so as to put the children in specific syndromic or nonsyndromic type of mental retardation. Down's syndrome and fragile X syndrome are commoner causes and relatively easily recognized. Physical measurements of height, weight, and head circumference should be taken. Neurological, dysmorphological hearing, and vision examination should be done. If head circumference is abnormal, parents head circumference should be taken and a pedigree chart made.
- Acquired causes and metabolic causes of mental retardation need to be excluded, particularly causes like neonatal hypothyroidism, phenyl ketonuria, high triiodothyronine level with SLC 16 A2 mutation, etc.
- A progressive form of mental retardation suggests metabolic cause and a gene mutation rather than chromosomal abnormality.
- If there are fetal neurological deficit or seizures, relevant imaging studies need to be done and sometimes specific abnormalities found in imaging studies may give away the mutation.
- The decision on neuroradiological and metabolic testing should be guided by clinical symptoms and inheritance pattern.
- Recurring risk for MR caused by single gene mutation should be determined according to mode of inheritance. This is essential for counseling the family.
- Finally genetic diagnostic work up in patients with mental retardation should ideally include karyotyping, fragile X mutation, CGH array, and sequencing of known MR genes.

We have a long way to go and we need many such studies from different areas of the country not only to understand the magnitude of the problem but also to develop our captivity to investigate such cases.
